# Medical resource consumption of moderate/severe psoriasis in a private health organization of Buenos Aires, Argentina^☆^^☆☆^

**DOI:** 10.1016/j.abd.2019.04.007

**Published:** 2019-12-06

**Authors:** María Laura Galimberti, Aldana S. Vacas, Barbara A. Hernández, María L. Bollea Garlatti, María J. Cura, Ricardo L. Galimberti

**Affiliations:** Dermatology Department, Hospital Italiano de Buenos Aires, Buenos Aires, Argentina

**Keywords:** Argentina, Cost of illness, Psoriasis

## Abstract

**Background:**

Despite the economic burden of psoriasis for patients and societies, scant information exists regarding the impact and burden of the disease in Argentina.

**Objective:**

The objective of this study was to estimate medical resource consumption and direct health care costs for patients with moderate/severe psoriasis in Buenos Aires, Argentina from the perspective of the payer.

**Methods:**

Adults with moderate/severe psoriasis (severity was defined as receiving systemic treatment), during January 2010–January 2014, aged 18 years and older, members of the Italian Hospital Medical Care Program with at least 18 months of follow-up were included. All data on hospitalizations, drug prescription, outpatient episodes, consultations, and investigations/tests in the 12 months before inclusion in the study were considered for the estimation of medical resource consumption and direct health care costs. First-quarter 2018 costs were obtained from the IHMCP and converted into US dollars (using the January 2018 exchange rate).

**Results:**

A total of 791 patients were included. The mean age at diagnosis was 34 ± 12 years. Almost 65% of the patients had a dermatologist as their usual source of care, 43% had internists, and 14% had rheumatologists. The average yearly direct cost was US$ 5326 (95% CI: 4125–7896) per patient per year.

**Study limitation:**

The single center design and the retrospective nature are the main limitations.

**Conclusion:**

This is the first Argentine study that evaluated the costs of moderate/severe psoriasis by taking into consideration the direct medical costs of the disease.

## Introduction

Psoriasis is a chronic inflammatory immune-mediated proliferative skin disorder that predominantly involves the skin, nails, and joints.^1^, ^2^

In 2014, Member States of the World Health Organization (WHO) recognized psoriasis as a serious non-communicable disease and adopted the resolution of the World Health Assembly (WHA), which calls for multilateral efforts to raise awareness and fight stigma. The worldwide prevalence of psoriasis is estimated to be approximately 1–3%.^2^, ^3^

Although psoriasis is not usually a life-threatening disease, several studies have shown that it has a significant impact on medical resource consumption in affected patients.^4^, ^5^

Despite the economic burden of psoriasis for the patient and for society as reported by several authors,^6^, ^7^, ^8^ scant information exists regarding the impact and burden of the disease in Argentina. To better understand this impact, the objective of this study was to estimate medical resource consumption and direct health care costs for patients with moderate/severe psoriasis in Buenos Aires, Argentina from the perspective of the payer.

## Methods

A retrospective, epidemiological, and observational study was conducted. The population studied included the members of the Italian Hospital Medical Care Program (IHMCP), a prepaid health maintenance organization. The IHMCP provides comprehensive medical and health services through two main hospitals and 24 medical offices to more than 164,456 members, primarily located in the urban areas within the autonomous city of Buenos Aires in Argentina. The city covers an area of 202 km^2^ and has a subtropical climate. It is located on the western bank of the Río de la Plata and has a population of 2,890,151.^9^ All patients who were diagnosed with moderate/severe psoriasis (severity was defined as receiving systemic treatment) during January 2010–January 2014, 18 years and older, who were members of the IHMCP and with at least 18 months of follow-up, were included in the study.^6^ Multiple methods of case retrieval were used to ensure complete ascertainment: (1) patients included in dermatology databases; (2) patients with the International Classification of Primary Care code in the IHMCP computer-based patient record system; and (3) patients with the International Classification of Diseases, Ninth Revision code on admission to hospital. Each potentially eligible subject underwent a standard procedure (clinical exam and biopsy) to confirm the diagnosis by dermatology experts. The study protocol and questionnaire were approved by the Ethics Committee before the initiation of the study.

### Resource utilization

Data on hospitalizations, drug prescription, out-patient episodes, consultations with general or specialist medical practitioners, and investigations/tests in the 18 months before the inclusion in the study were collected from electronic records of the included patients. Electronic records of IHMCP patients provide daily routine information on their diseases and therapies.

### Costs

Costs were calculated using the bottom-up approach and grouped by perspective. To obtain the annual cost per patient per year, annualized resources were multiplied by the irrespective unit costs. January 2018 unit costs were derived from the IHMCP. The unit costs in Argentine pesos were converted to US dollars using the January 2, 2018 exchange rate (US$1 = AR $19.23). Unit costs derived from the IHMCP were compared with those from the public sources of Argentina, including the National institute of Statistics and Surveys,^9^ the National Cost Database (Nomenclador Nacional),^10^ and the Public Price List of Drugs^11^ to avoid significant differences. For the direct analysis cost, systemic therapy was stratified into the following: (a) biologic systemic treatment; (b) non-biologic systemic treatment; and (c) other systemic treatment that included UV and PUVA therapy. Unit costs are shown in table 1.

**Table 1:** Unit costs in US dollars.

Unit costs	US dollars
Specialist consultation, dermatology	36
General consultation	26
Specialist consultation, rheumatology	34
Emergency room care (day)	110
Hospital admissions (day)	186
PUVA therapy (per cycle of 24 sessions)	1745
UVB (per cycle of 24 sessions)	2153
Cyclosporine (monthly costs)	312
Methotrexate (monthly costs)	26
Acitretin (monthly costs)	74
Etanercept (monthly costs)	2398
Adalimumab (monthly costs)	2389
Ustekinumab (monthly costs)	2480
Infliximab (monthly costs)	3968
Certolizumab (monthly costs)	2294
Golimumab (monthly costs)	2250

### Statistical analysis

Patients’ demographic and disease characteristics, as well as resource utilization and costs, were analyzed using descriptive statistics (percentage, mean, and SD). Confidence intervals (95%) were estimated by using non-parametric bootstrapping. A non-parametric statistical test (Wilcoxon–Mann–Whitney test) for independent samples was used to determine whether differences between comparisons performed were statistically significant (*p* < 0.05). Stata v. 10.1 software (Stata Corp. – College Station, TX, United States) was used for the analysis.

## Results

### Patient characteristics

A total of 791 patients were included in the study. The mean age at diagnosis was 34 ± 12 years, 49% were women, and the mean disease duration was 10 ± 16 years. Other patient characteristics are presented in table 2.

**Table 2:** Baseline characteristics of patients enrolled.

*n*	791
Male sex	387
Female sex	404
Mean age (years ± SD)	34 ± 12
Years since diagnosis, SD	10 ± 16
Palmoplantar, *n* (%)	31 (4)
Guttate, *n* (%)	55 (7)
Plaques, *n* (%)	672 (85)
Pustulosa, *n* (%)	16 (2)
Erythrodermic, *n* (%)	16 (2)

SD, standard deviation.

The patients were treated with biologic and non-biologic medications. Patients are presented descriptively in table 3.

**Table 3:** Treatment received by included patients.

	*n*	%
Patients who received systemic biologic treatment	99	12.5
Patients who received systemic non-biologic treatment	675	85
Patients who received other systemic treatment (PUVA and UVB)	323	41

### Resource utilization

Almost 65% of the patients had dermatologist as their usual source of care, 43% had internists, and 14% had rheumatologists. Only 8% of the patients had had an emergency room admission to manage psoriasis symptoms. Data on other resource utilization by patients are presented in table 4.

**Table 4:** Resource utilization and annual direct medical costs of included patients (*n* = 791).

*n* = 791					
	Resource consumption			Annual direct medical costs	
Resources	Unit	% (*n*) of resource utilization^a^	Mean ± SD of resource utilization^a^	Mean	95% CI
Specialist consultation, dermatology	Visits	65 (1536)	0.7 (0.3)	16.3	9–21.2
General consultation	Visits	47 (1583)	1.01 (0.6)	12.3	8.5–15.5
Specialist consultation, rheumatology	Visits	14 (331)	0.1 (0.01)	5.2	3.1–7.2
Emergency room care	Visits	8 (189)	0.09 (0.01)	2.5	1.1–3.7
Hospital admissions	Days	14 (330)	0.08 (0.009)	4.1	2.1–5.8
Systemic biologic treatment	Patients	12.5 (99)	NA	3450	3113–4560
Systemic non-biologic treatment	Patients	85 (675)	NA	1097	867–1234
Other systemic treatment (PUVA and UVB)	Patients	41 (323)	NA	867	1123–1879
Total direct medical costs (US $)	–	–	–	5326	4125–7896

a Resource utilization is by patient per year. CI, confidence interval; NA, not applicable.

### Costs

Direct costs were obtained from resource consumption, as previously described. The most relevant source of costs was the medical treatment received, followed by physician visits. The average yearly direct cost per patient was US$ 5326 (95% CI: 4125–7896).

All costs obtained from resource utilization are presented in table 4.

## Discussion

With the introduction of biologics over the past years, there has been a marked shift in treatment modalities that has resulted in a great need for the updating of costs.^8^ This is the first study performed in Argentina and in the region that evaluates the costs of psoriasis by taking into consideration the direct medical costs of the disease in the moderate-to-severe stage. Mean annual cost per patient was estimated at US$ 5326.

This study is consistent with previous cost-of-illness studies performed in Germany and some European countries.^6^, ^8^, ^12^ In Germany, Jungen et al. evaluated primary data on 1158 patients collected in a real care setting (a random sample from 132 centers across Germany).^8^ The mean cost was € 5543 (median € 2030).^8^ The total costs found by other authors range from € 1079 per patient per year in Spain, from € 387 to € 6398 in Switzerland (including biologics), and € 11,434 in Italy (PASI > 20).^13^ Variations in costs can be explained by year of data, differences in health care systems (especially pricing of medication), and methodological differences such as payer perspective and severity of disease.^13^ In Germany, in moderate-to-severe plaque-type psoriasis, the out-of-pocket costs were estimated to be € 794 per year on average (15% of the mean total costs of that study).^6^ Berger et al. estimated € 562 (20% of the mean total costs of that study).^14^ Another study performed in nine states of the United States, which included 200 patients with moderate-to-severe psoriasis and evaluated health care resources for six months, showed that the direct cost in that population was US$ 11,291 and indirect costs were US$ 2101.^12^ In that group of patients, 79.5% were prescribed one or more biologic medications for psoriasis to control the disease.^12^

The present study has many limitations. First, the sample involves patients from only one center. Therefore, a larger sample that involves more centers would be necessary to generalize the results. The second limitation is the way the information was obtained (retrospectively, by collecting information from medical records). Nevertheless, considering that the patients from the IHMCP are seen solely in its own centers and that all medical records are registered, the possibility of bias is low. The third limitation is that indirect costs were not obtained.

In Argentina, this is the first study designed to capture the economic burden of the disease. The important and novel information provided about the disease-related economic burden is the main contribution of this study to the existing literature for psoriasis both in Argentina and the region in terms of costs in a developing country. This information is a valuable tool for relevant and wise decision-making in the field by understanding the exact behavior of the disease in the local setting, and is supplementary to the use of information from Europe and North America, where both resource utilization and costs may be quite different from those observed in a developing country like Argentina.^2^, ^7^

## Conclusion

In conclusion, the study provides important information about the economic burden of the disease in Argentina. Future research performed here as well as in other countries of the region, taking into consideration direct and indirect costs of the disease, will expand knowledge and improve management of the disease in the region, an issue of great importance.

## Financial support

None declared.

## Authors’ contribution

María Laura Galimberti: Approval of the final version of the manuscript; conception and planning of the study; elaboration and writing of the manuscript; obtaining, analyzing, and interpreting the data; effective participation in research orientation; intellectual participation in propaedeutic and/or therapeutic conduct of the cases studied; critical review of the literature; critical review of the manuscript.

Aldana S. Vacas: Approval of the final version of the manuscript; obtaining, analyzing, and interpreting the data; critical review of the literature.

Barbara A. Hernández: Approval of the final version of the manuscript; obtaining, analyzing, and interpreting the data; critical review of the literature.

María L. Bollea Garlatti: Approval of the final version of the manuscript; obtaining, analyzing, and interpreting the data; critical review of the literature.

María J. Cura: Approval of the final version of the manuscript; obtaining, analyzing, and interpreting the data; critical review of the literature.

Ricardo L Galimberti: Approval of the final version of the manuscript; obtaining, analyzing, and interpreting the data; critical review of the literature.

## Conflicts of interest

None declared.
